# Production of Iodine in France

**Published:** 1874-04

**Authors:** 


### THE PRODUCTION OF IODINE IN FRANCE.

From Prof. Beilstein's report on the Great Chemical Industries at the Vienna Exposition we extract the following in regard to the manufacture of iodine and its compounds:

The crude material which is used in France for obtaining iodine is sea weed, which grows on the low ands of the coasts of Normandy, Brittany, Ireland and Scotland, which are under water at high tide, but out of water at low tide. These weeds are collected twice a year, in March and October. Owing to the scarcity of fuel in these parts, the weeds were very early used for heating purposes, as also for fertilizers. Toward the end of the 17th century a systematic burning of the weeds began, which was done then as now, twice a year, in round or rectangular pits in the open air on the sea-shore. The wrack soda, or kelp, thus obtained was employed for making glass until the discovery of another method of making soda Another use was therefore sought or kelp, and in 1798 a manufactory conducted by Couturier was started at Cherbourg, for the purpose of separating the different kinds of kelp, in order to furnish the glass makers with a better material. In 1811, a saltpetre manufacturer in Paris, named Courtois, discovered iodine in the mother liquid of these salts. In 1824, Tissier, of Cherbourg, founded the first works for manufacturing iodine. Soon after, in 1827, he erected a second manufactory there for Couturier, and in 1829 the two were united under the firm of Cournerie et fils In 1830, Tissier founded an iodine factory in Conquet, which is now the most important of all, the annual production being sixteen to eighteen tons of iodine and iodide of potassium. Since that time the number of factories has increased to nine.

The nine firms mentioned annually consume 12,000 tons of crude kelp, obtained from 540,000 tons of green sea weeds, and produce 2,400 tons of saltpetre, 2,000 tons of chloride of potassium, 1,800 tons of common salt, 1,520 tons sulphate of potash, 120 tons Glauber salt, 40 tons pure iodine, 4 tons bromine and 15 tons sulphur. The residue, when dry, contains 22.4 per cent carbonate of lime and 9.4 per cent sulphate of lime, and is employed as a fertilizer.

The improvements claimed by the exhibitors in this department are, calcining the plants in continuously-operating, closed furnaces, and the precipitation of iodine by means of the oxygen of the air.—*Jour. of Applied Chemistry.*

